# Biophysical Properties of Optogenetic Tools and Their Application for Vision Restoration Approaches

**DOI:** 10.3389/fnsys.2016.00074

**Published:** 2016-09-02

**Authors:** Simon D. Klapper, Anka Swiersy, Ernst Bamberg, Volker Busskamp

**Affiliations:** ^1^Center for Regenerative Therapies Dresden, Technische Universität DresdenDresden, Germany; ^2^Max Planck Institute of BiophysicsFrankfurt, Germany

**Keywords:** optogenetics, channelrhodopsin, halorhodopsin, retinal degeneration, retinitis pigmentosa

## Abstract

Optogenetics is the use of genetically encoded light-activated proteins to manipulate cells in a minimally invasive way using light. The most prominent example is channelrhodopsin-2 (ChR2), which allows the activation of electrically excitable cells via light-dependent depolarization. The combination of ChR2 with hyperpolarizing-light-driven ion pumps such as the Cl^−^ pump halorhodopsin (NpHR) enables multimodal remote control of neuronal cells in culture, tissue, and living animals. Very soon, it became obvious that this method offers a chance of gene therapy for many diseases affecting vision. Here, we will give a brief introduction to retinal function and retinal diseases; optogenetic vision restoration strategies will be highlighted. We will discuss the functional and structural properties of rhodopsin-based optogenetic tools and analyze the potential for the application of vision restoration.

## Retinal Structure, Organization, and Signal Processing

**Figure 1:**
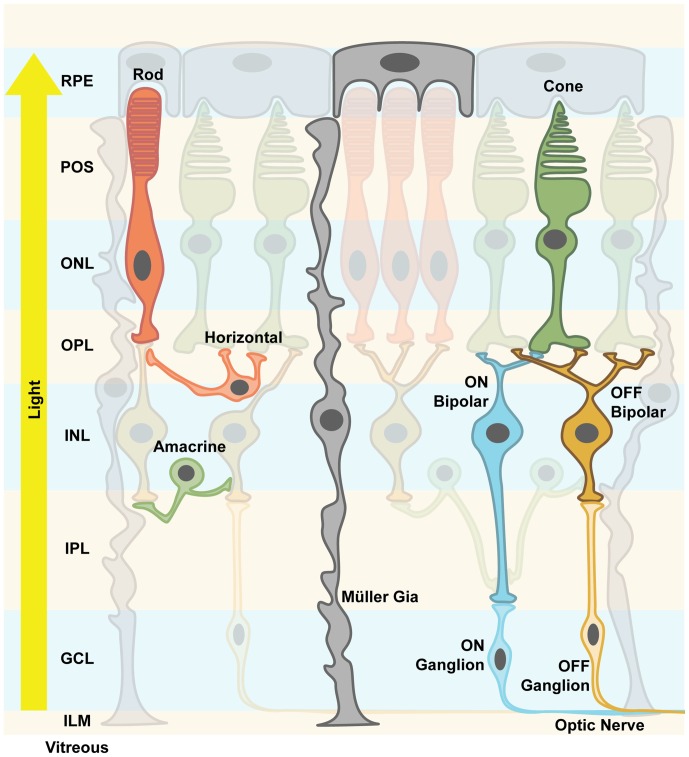
**Retinal structure.** The vertebrate retina is a layered structure. The retinal pigment epithelium (RPE) cells send their apical processes into the photoreceptor outer segment (POS) layer. The photoreceptors are densely packed and the layer containing their nuclei is called outer nuclear layer (ONL). Axons spread from there to the outer plexiform layer (OPL), where the synapses between photoreceptors, excitatory bipolar cells and inhibitory horizontal cells are formed. Along with the amacrine cells, their cell bodies and nuclei are located in the inner nuclear layer (INL). Amacrine cells create inhibitory synapses onto bipolar cells’ axons. Bipolar cells either depolarize their respective ganglion cells upon light stimulus (ON channel) or after the light stimulus (OFF channel). Inside the inner plexiform layer (IPL), this precisely processed signal is finally processed and output via the axons of ganglion cells, which send action potentials along the optic nerve to higher brain areas. Mixed ON/OFF channels also exist. Müller glia cells support neuronal functions and mediate important signals, e.g., immune responses. They are considered more plastic, and spread their processes all over the retinal structure from the RPE to the inner limiting membrane (ILM). The light travels from the pupil through the vitreous, hitting first the processing neuronal network before reaching the actual photoreceptors, hence the notion of an “inverted eye” in vertebrates.

The mammalian retina is a layered structure that sits at the back of the eyeball. It consists of five neuronal cell classes (Masland, 2001; Nguyen-Ba-Charvet and Chédotal, 2014) and one major glial cell type, the so-called Müller cells (Reichenbach and Bringmann, 2013). The distal layer is called the outer nuclear layer (ONL) and it contains the cell bodies of rod and cone photoreceptors. The inner nuclear layer (INL) consists of excitatory bipolar cells, as well as inhibitory horizontal and amacrine cells. The ganglion cell layer (GCL) consists of ganglion cells: their axons are bundled to form the optic nerve and relay light information to higher brain areas. Retinal neurons are connected in two plexiform layers, the inner and outer plexiform layers (OPL), where most of signal processing by inhibitory neurons takes place (Gollisch and Meister, 2010; Figure 1). Of note is the inverse layered orientation: photoreceptors face inwards. As a consequence, light passes through the entire retinal tissue before it can be detected by photoreceptors, with the notable exception of the fovea. In the OPL, horizontal cells mediate lateral inhibition to precise visual signals. Amacrine cell interactions in the inner plexiform layer (IPL) add multiple modes of visual processing, for example direction selectivity. In addition, downstream of the photoreceptors, two major, morphologically distinct pathways diverge, the ON and the OFF pathways, which are activated by increments or decrements of light, respectively. ON and OFF bipolar cells project onto corresponding retinal ganglion cells. It is thought that each aspect of the visual scene is transmitted via different parallel channels that are formed by different ganglion cell types. It has been shown that there are at least 30 different channels in mice (Roska and Werblin, 2001; Baden et al., 2016). In summary, the retina is not only a light-receiving element of the brain, but a tiny sophisticated biological computer that first converts photons into electrical signals then processes them before the information is sent to higher brain areas.

## Photoreceptor Functions and Retinal Degeneration Diseases

**Figure 2:**
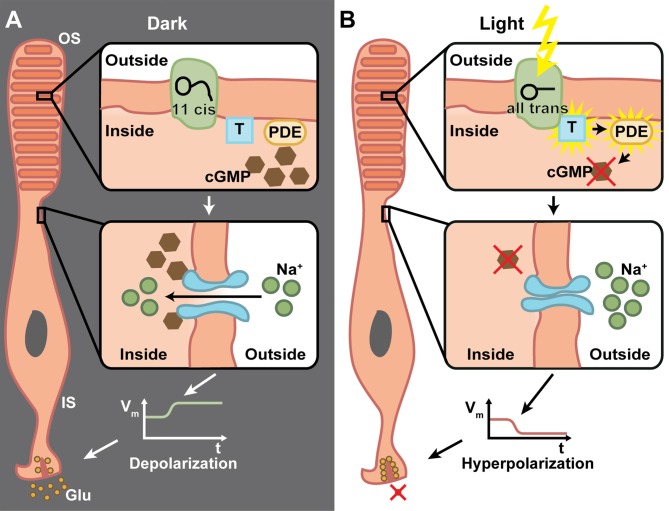
**The phototransduction cascade in vertebrate photoreceptors. (A)** In the dark, 11-cis retinal is bound to rhodopsin, which is located inside the membrane of the outer segment’s (OS) discs. The G-protein transducin (T) and phosphodiestherase (PDE) are inactive (upper panel). Cyclic guanosine monophosphate (cGMP) triggers the opening of cation channels in the photoreceptor membrane, which mediate a Na^+^ influx (middle panel). This in turn depolarizes the membrane potential of the inner segment (IS) and triggers the release of glutamate (Glu) from ribbon synapses (lower panel). **(B)** Upon light absorption, 11-cis retinal is converted to all-trans retinal and dissociates from the rhodopsin. This activates the transducin, which in turn activates PDE, whose catalytic activity degrades cGMP (upper panel). This effectively lowers the cGMP concentration, which leads to closure of cation channels (middle panel). Thus, the IS is hyperpolarized and the Glu exocytosis is stopped (lower panel). Due to the G-protein involved in the transduction, a very high amplification of single photon responses is possible.

A common feature of retinal degenerative diseases is the progressive loss of photoreceptors (Bramall et al., 2010). As in many other species, humans have two types of photoreceptors for image-forming vision, the highly sensitive rods important for low light vision and the less sensitive cones for high acuity daytime and color vision (Dowling, 1970). There are three cone subtypes, which mainly differ in the spectral tuning of their photopigments, cone opsins, and thereby facilitate trichromatic vision (Wald, 1964). Cone opsins and rhodopsins in rod photoreceptors are G-protein-coupled receptors (GPCRs) that convert light into electrochemical signals via a G-protein cascade that finally leads to a hyperpolarization of the photoreceptor cell (Figure 2). This process is called phototransduction and it takes place in specialized cellular compartments, the outer segments (OS) that are surrounded by retinal pigment epithelium (RPE) cells (Koch and Dell’Orco, 2015). Neuronal activity in general is mediated via the depolarization of the membrane potential, whereas hyperpolarization results in neuronal inhibition. Therefore, it is of note that photoreceptors are active in the dark and release the neurotransmitter glutamate to the synaptic cleft, whereas light-induced hyperpolarization stops the glutamate secretion to which the postsynaptic bipolar and horizontal cells respond (Heidelberger et al., 2005).

Cone photoreceptors are not evenly distributed over the entire retina. These cells are clustered in the macula, of which the central area called the fovea comprises the spot of highest visual acuity (Provis et al., 1998). The fovea consists only of cone photoreceptors: these project onto sideward-oriented downstream retinal cells. Rodents lack a macula and therefore, are not well suited for modeling human cone diseases such as age-related macular degeneration (AMD; Mehta, 2015). Nevertheless, there are many adequate mouse models for rod diseases such as retinitis pigmentosa (RP). In RP, rods are affected by mutations leading to night blindness (Hartong et al., 2006). Later on, these cells degenerate and the death zone progresses from the periphery towards the retinal center, which results in tunnel vision. In the late stages, cone photoreceptors lose their OS, become light insensitive and to some degree, also degenerate, causing overall blindness. RP phenotypes, their onset and progression are highly variable among patients, also depending on the type of genetic mutation (Jacobson et al., 2010). To date, more than 90 mutations causing RP have been identified and mapped^1^. Thus, blindness is a consequence of functional impairments and degenerated photoreceptor cells.

## Therapeutic Strategies

Therapeutic interventions in degenerative diseases are a race against time: it is necessary to intervene before degeneration occurs. To date, treatments that reverse vision loss are still in pioneering phase. For example, one of the most promising examples is the RPE-65 gene transfer that is targeted to RPE cells in Leber congenital aumorosis (LCA; Testa et al., 2013; Weleber et al., 2016). In gene therapy approaches, the transfer of a corrected copy of a disease-causing gene is limited by the disease phenotype (it must be slow and recessive) and by the size of the gene. The gold standard for ocular gene transfer uses adeno-associated viruses (AAV) that can carry about 4.7 kb of single-stranded DNA (Grieger and Samulski, 2005). AAV particles are relatively small (20 nm in diameter) and the viral single-stranded DNA genome is encapsulated in capsids, which classifies their serotype. To date, there are different serotypes in use, which target different cell surface receptors. Therefore, this tropism can be adjusted to transfect preferred cell types (Grieger and Samulski, 2005; Ojala et al., 2015; Salganik et al., 2015). Therapeutic interventions for dominant mutations additionally need a cellular depletion of the mutated endogenous copy. In addition, interventions to stop or delay the disease progression by applying neuroprotective factors (Trifunović et al., 2012; Froger et al., 2014; Léveillard et al., 2014) as well as substituting degenerated photoreceptors using cell transplantations (Jayakody et al., 2015) are also being tested extensively.

In this review, we will focus on optogenetic interventions. An optogene confers light sensitivity to any targeted neuron and thereby functionally turns it into artificial photoreceptors (Fenno et al., 2011). Hence, applying optogenetic tools to the remaining retinal neurons after the degeneration or dysfunction of intrinsic photoreceptors was the obvious step. This approach is mutation-independent and it is suited for patients that lack or have non-functional photoreceptors and are therefore legally blind. Still, a substantial number of other retinal cell types has to be intact for optogenetic targeting. We will highlight approaches that have the potential to be tested, and some that are already being evaluated for clinical trials, namely those where the optogenes fit into recombinant AAV vectors. In addition to the optogene, suitable promoter elements that mediate sufficient optogene expression into the recombinant AAV genome are necessary. These promoters are either cell-type-specific or mediate ubiquitous expression. The choice depends on the availability and strength of suitable elements, and the anticipated strategy of the targeted cell types. In a laboratory setting, researchers can control transgene expression via genetic tricks such as the Cre-LoxP system, which is not applicable in therapeutic approaches. Although there is a substantial body of interesting optogenetic work in transgenic mice (Vann and Xiong, 2016), here we will mostly focus on strategies that rely on AAV transfer.

## The Biophysics of Optogenetics

**Figure 3:**
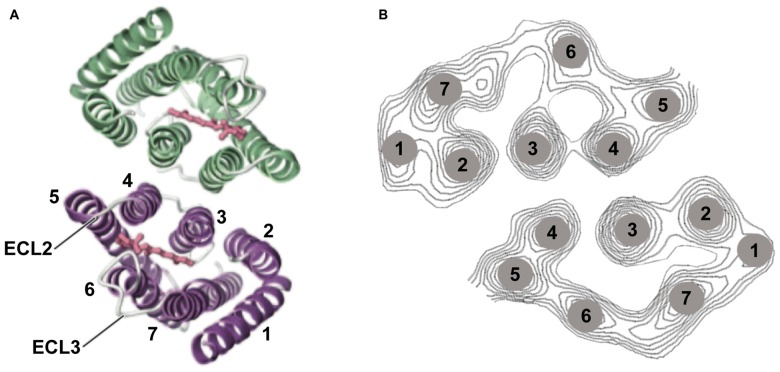
**Channelrhodopsin-2 (ChR2) structure. (A)** Structural analysis using 2D and 3D crystallography has revealed that ChR2 appears as a dimer. The channel-forming helices are helices 1, 2, 3, and 7 and two extracellular loops (ECL) are highlighted (Müller et al., 2011, 2015; Kato et al., 2012). **(B)** Helix movement after light excitation of helices 2 and 7 (part of the cation pathway) was observed, as well as movement of helix. Panel **(A)** was modified from Kato et al. (2012) and **(B)** from Müller et al. (2015).

Especially when applying optogenetic tools to therapeutic interventions, it is of high importance to understand the strengths and limitations of microbial opsins. Here we present the biophysical properties of the most important optogenetic tools applied in neurobiology, with a focus on ophthalmic diseases. We describe the molecular properties, important for determining the appropriate application for vision restoration. Two classes of microbial rhodopsin-based tools are used: light-gated ion channels, named channelrhodopsins (ChR; Nagel et al., 2003) and light-driven ion pumps such as the Cl^−^ pump halorhodopsin (NpHR; Duschl et al., 1988; Bamberg et al., 1993) or the H^+^ pump Arch (Chow et al., 2010). Light-gated anion channels also exist, both engineered (Berndt et al., 2014; Wietek et al., 2014) or discovered in algae (Govorunova et al., 2015; Wietek et al., 2016). However, these hyperpolarizing anion channels have not yet been applied to confer light sensitivity to blind retinas. Major points are light sensitivity and single-channel parameters such as conductance and the lifetime of the channel. All rhodopsins consist of a seven-transmembrane helix motif, whereby the chromophore retinal is bound via a Schiff base to the peptide chain of helix 7 (Figure 3). Rhodopsins undergo a photocycle after light absorption. The photocycle kinetics control the channel or pump activity. In the case of the visual rhodopsins, the enzymatic cascade that yields the hyperpolarization of the cells determines the speed of activation.

## Channelrhodopsin-2

Channelrhodopsin-2 (ChR2) is present in the “eyespot” of the unicellular alga *Chlamydomonas reinhardtii* and its natural function is connected with phototaxis to help the organism obtain optimal light conditions (Sineshchekov et al., 2002). ChR2 is a light-gated cation channel with a high permeability for protons, 10^6^ times larger than its permeability for monovalent cations Na^+^ and K^+^ (Nagel et al., 2003). There is also a minor permeability for Ca^2+^. At physiological pH, however, the proton conductance can be ignored due to the low [H^+^]. In addition, ChR2 is a leaky, outwardly directed proton pump, whereby the leak actually represents the natural channel function (Feldbauer et al., 2009). The single channel parameters were determined by noise analysis as single channel conductance γ ≈ 80 fS and open probability *P*_o_ ≈ 0.5 at room temperature and physiological ion concentrations. These properties allow the determination of the number of active ChR2 copies per cell in optogenetic experiments in which *n* ≈ *I*_ph_ / γ*P*_o_, where *I*_ph_ represents the measured photocurrent.

**Figure 4:**
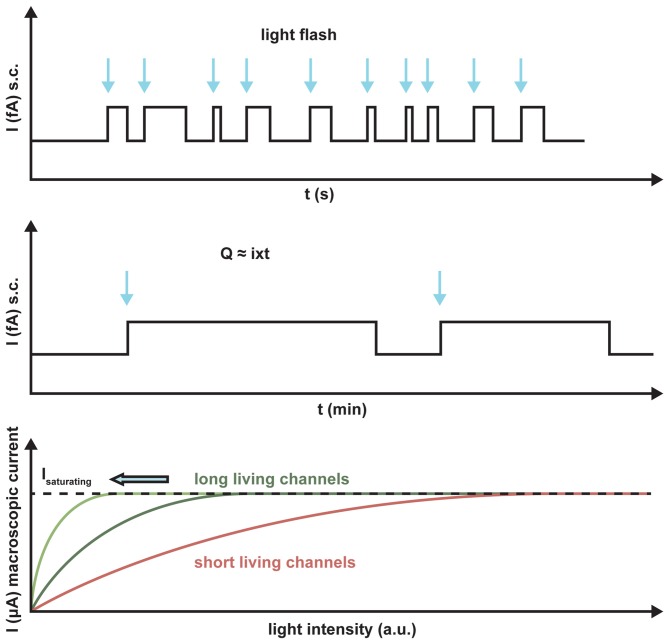
**Schematic representation of the light sensitivity of ChR2 variants with different lifetimes.** The arrows indicate the light activation of the putative single channels. The longer the lifetime, the more charge Q will be transported per absorbed photon, I is the single channel current. Light saturation is reached at much lower light levels for the long-lasting channels than for short-term channels, as demonstrated by the number of absorbed photons.

ChR2 maximally absorbs blue light at 470 nm. At this wavelength, the penetration into the tissue is poor as compared to the recently published red light absorbing ChR Chrimson with a peak of action at 590 nm because of the scattered light and light absorption in the tissue (Klapoetke et al., 2014). In addition, light sensitivity and frequency response are important in the retina for visual restoration. Depending on the transduced cell type, frequencies between 30 and 800 Hz are necessary for optimal activation. This implies that a shorter lifetime of the channel results in a better frequency response of the neuronal cell. Because of smaller ion translocation, the light sensitivity is drastically reduced in a fast ChR, whereas an increased channel lifetime yields an increased light sensitivity. A typical quantum efficiency of rhodopsin-like actuators is around 0.5, so that manipulation attempts to increase this value can only lead to small increases in light sensitivity. Only a higher ChR expression in the membrane can increase the sensitivity, because all the single-channel conductances of different ChRs tested so far are almost invariant (Figure 4).

A drawback of using microbial opsins is the dynamic range of applied light intensity, which usually does not exceed more than one order of magnitude with respect to the absorbed photons. Compared to the intact visual system which senses light over nine orders of magnitude, this means relatively high light intensities have to be applied to elicit a reasonable light response in the mammalian retina, especially for the fast ChR2 derivates. In other words, low ambient light must be amplified, whereas high light intensities have to be reduced. To achieve this in a therapeutic context, a goggle has to be developed that consists of a digital camera and a photodiode array (Cepko, 2010). The device allows the camera image to be transformed into a constant high photon density representation to the transduced cells in the retina. Due to high light sensitivity of the digital cameras within such a device, light intensities of up to five orders of magnitude could be registered by the transduced retinal cells.

## Light-Driven Ion Pumps

Halorhodopsin from *Natronomonas pharaonis* (NpHR) is a light-driven inwardly-directed Cl^−^ pump, which hyperpolarizes the archaebacterial cell by up to −200 mV (Duschl et al., 1988; Bamberg et al., 1993). The resulting electrochemical gradient is used for ATP synthesis and for the activation of secondary transporters in the cell membrane. NpHR undergoes a photocycle, where the Cl^−^ transport is tightly coupled to its kinetics, i.e., one unit charge per successful photocycle.

**Figure 5:**
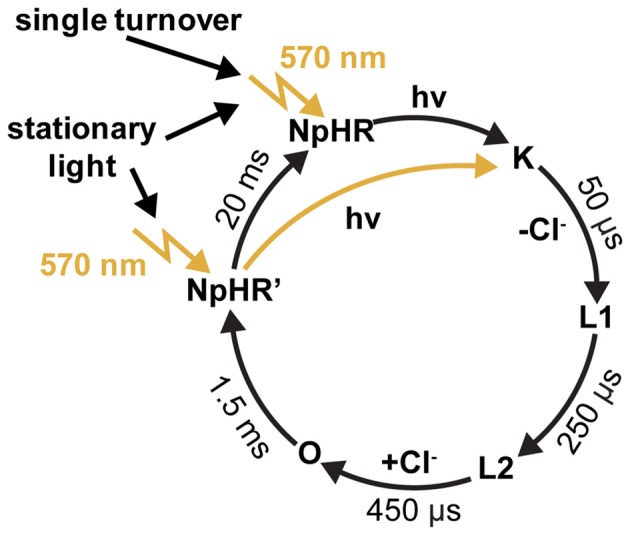
**The photocycle of halorhodpsin (NpHR).** Flash photolysis experiments revealed a dark-adapted and a light-adapted ground state, with identical absorption maxima at 570 nm (Chizhov and Engelhard, 2001).

NpHR was the first light-driven ion pump applied in neuronal cells (Han and Boyden, 2007; Zhang et al., 2007). It has been shown in an electrophysiological study that, after the sequential absorption of two photons, the photocycle of NpHR is short-circuited (Figure 5). This implies that the rate-limiting step of the photocycle lasts 1.5 ms, which corresponds to a turnover of 700 ions per second. It has also been shown that the proton pump Arch has similar properties (Chow et al., 2010; Kleinlogel et al., 2011b). Compared to activated wildtype ChR2 with a lifetime of 10 ms, which transports 10,000 unit charges per second under physiological conditions, the NpHR performance is only 10 times less (Feldbauer et al., 2009). Depending on the expression level, a short light pulse which initiates a single turnover of the rhodopsins is sufficient for the activation or inactivation of neuronal cells by ChRs or the light-driven ion pumps (Kleinlogel et al., 2011b). These properties make these fast cycling ion pumps valuable tools for the hyperpolarization of residual cone cells as demonstrated in retinal photoreceptors (Busskamp et al., 2010; Chuong et al., 2014).

## Rhodopsins and Engineered G-Protein-Coupled Receptors

Very recently, alternate optogenetic approaches using human rhodopsin or rhodopsin-like GPCRs have been applied. By this procedure these intrinsic photoreceptor molecules could be functionally expressed in other retinal cell types in photoreceptor-deficient mouse retinas. This approach certainly has potential, because these light-sensitive proteins activate endogenous signal transduction cascades after light flashes. This amplification step results in light sensitivities, several orders of magnitude higher than those of the microbial rhodopsins (Cehajic-Kapetanovic et al., 2015; Gaub et al., 2015; van Wyk et al., 2015).

## Optogenetic Vision Restoration Strategies

After the pioneering optogenetic publication in 2005 (Boyden et al., 2005), two different vision restoration strategies were described. First, an approach to target as many retinal cell types as possible, mostly ganglion and amacrine cells (Bi et al., 2006). In this approach, ON and OFF pathways would be phased to ON, and other retinal processing would be lost. It was assumed that the brain is plastic enough to extract and process the visual input. Second, a cell-type-specific approach was applied to target ON bipolar cells, aiming to restore inner retinal signal processing features (Lagali et al., 2008). We now know from electronic prosthesis, transplantation experiments that patients are able to recognize letters and primitive visual schemes upon non-cell type specific electrical stimulation (Stingl et al., 2015; Luo and da Cruz, 2016). This indicates that a random retinal cell-targeting approach will likely restore image-forming vision. Nevertheless, whenever retinal cell types that are more upstream, such as the remaining cone photoreceptors, and bipolar and amacrine cells, can be targeted, retinal processing features will also be restored. This will likely improve the quality of restored vision, but ultimate proof will only come when the first treated patients are tested.

Cell-type-specific approaches rely on promoter elements that drive transgene expression exclusively in the target cell type. For AAV-mediated optogene expression, small promoter elements that provide relatively strong expression are needed; sufficient amounts of proteins are also needed to cover the cell’s surface membrane. The biggest limitation for retinal gene transfer is the lack of specific and strong promoter elements to target individual cell types in the retina. However, controlling expression in photoreceptors and ON bipolar cells is already very efficient (Li et al., 2008; Dalkara et al., 2013; Cronin et al., 2014) and it is only a matter of time before promoter elements for other cells of interest are identified and used. To date, there are many unspecific promoter elements such as the chicken beta-actin (CAG; Miyazaki et al., 1989) or cytomegalovirus (CMV) promoter (Du et al., 1996) that provide strong gene expression in multiple cell types and fit to recombinant AAV genomes.

## Targeting Ganglion Cells

**Figure 6:**
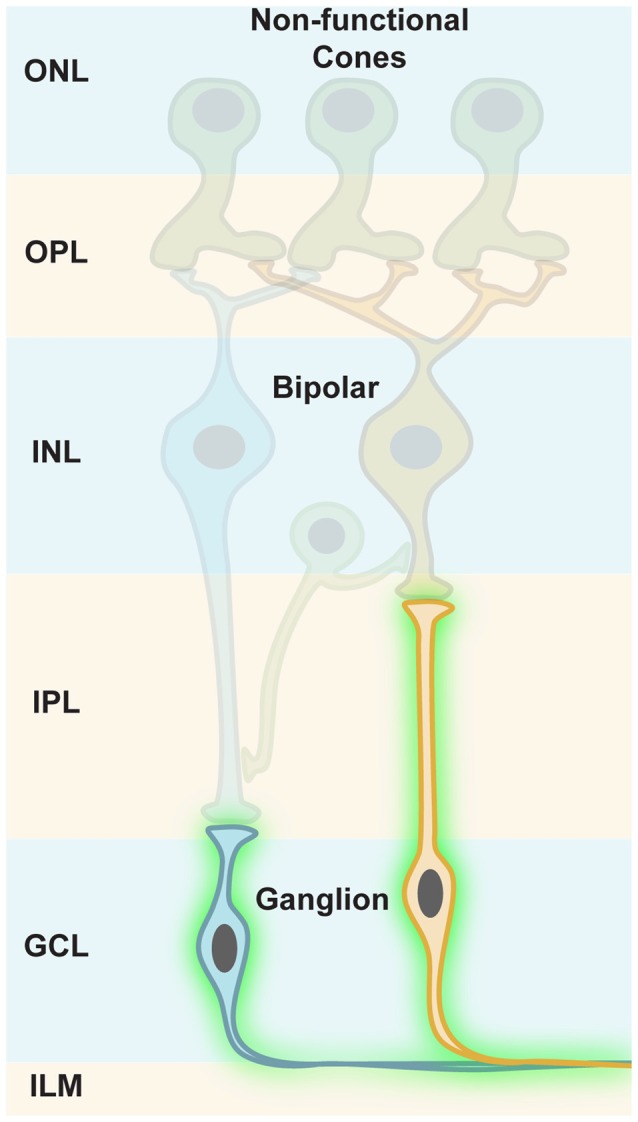
**Targeting retinal ganglion cells.** In photoreceptor diseases (e.g., retinitis pigmentosa, RP), cones lose their light-sensitive outer segments (OS) and their cell bodies merge with the INL, but ganglion cells persist in blind retinas. Along with their susceptibility to viral gene transfer through the vitreous, this makes ganglion cells an ideal cellular target for optogenetic restoration of visual function.

Ganglion cells are the retinal output cells and their axons relay visual information to higher brain areas. Upon photoreceptor degeneration, ganglion cells persist in blind retinas and only modest remodeling is reported (Marc et al., 2003; Jones et al., 2016). Due to their persistence during disease, ganglion cells are ideal cellular targets for optogenetic restoration of visual function (Figure 6). However, it is notable that there are no specific promoter elements that are only active in ON, OFF, or ON-OFF ganglion cells for AAV gene transfer. Therefore, it is necessary to apply ubiquitous promoters, which would phase the ON, OFF and ON-OFF ganglion cells to ON channels when using a depolarizing optogene. This means that upstream retinal processing would be lost. As mentioned above, the plasticity of higher brain areas likely compensates for some loss of quality in the retinal output signals. Additionally, specific promoter elements targeting ganglion cell subpopulations will probably be developed in the future.

Ganglion cells are located in the ganglion cell layer (GCL), adjacent to the vitreous body (Figure 6). In rodents, AAV administration into the vitreous space leads to efficient infection of ganglion cells, specifically in mouse models, whereas in primates the inner limiting membrane (ILM) resembles a barrier for AAV particles. The infected cells mostly cluster around the foveal rim (Vandenberghe et al., 2013). A pan-retinal transfection of ganglion cells would require additional removal of the ILM, or would need recombinant AAV capsids that can transcend it (Dalkara et al., 2009). Therefore, this difference in AAV gene transfer between mice and primates highlights another obstacle that has to be overcomed during the translational process.

Pioneering optogenetic vision restoration work was performed in the Pan lab in 2006 (Bi et al., 2006). Bi et al. (2006) successfully delivered ChR2 that was driven by a CAG promoter and fused to green fluorescent protein (GFP) via AAVs to inner retinal neurons, mostly ganglion cells, of blind rd1 mice. Thereby, robust ON responses were restored and transmitted to higher brain areas, as demonstrated by measured visual evoked potentials (VEP) in the visual cortex. Stable ChR2 expression and function in ganglion cells could be detected in 18-month-old rd1 mice without detectable toxicity, suggesting that long-term ChR2 expression in mouse ganglion cells is safe (Ivanova and Pan, 2009). Applying more sensitive ChR2 derivates led to improved light sensitivity in mouse ganglion cells (Pan et al., 2014).

This approach was further successfully tested in ganglion cells of blind Royal College of Surgeons (RCS) rats, resulting in reactivated ganglion cell spike responses, VEPs, and improvements in visually guided behavioral tests (Tomita et al., 2010). It is of note that AAV-mediated delivery of ChR2 in younger animals was more effective than in aged rats with progressed retinal degeneration (Isago et al., 2012). Surprisingly, the subretinal delivery of AAVs also resulted in ChR2 expression in non-retinal organs such as the heart, lung, and intestine (Sugano et al., 2011). These data suggest that AAV particles can exit the eyeball, remain infectious, and cause unwanted ChR2 expression in other organs. Instead of using the CAG promoter, a neuron-specific promoter element would likely omit expression in non-neuronal cell types. Still, the overall immune response against the viral capsids and ChR2 protein was extremely mild, suggesting that long-term ChR2 expression was well tolerated in rats (Sugano et al., 2011). It was also shown that a modified *Volvox* channelrhodopsin-1 (mVChR1) expressed in ganglion cells conferred a broad-spectrum light sensitivity to former blind rats, resulting in VEPs and visually guided behavioral test improvements (Tomita et al., 2014). The usage of more red-shifted depolarizing optogenetic tools (Chuong et al., 2014; Klapoetke et al., 2014) is favorable because the 470 nm light needed to activate ChR2 can be phototoxic, as high intensities are needed. Furthermore, blue light also triggers the pupillary light reflex that is triggered by melanopsin-expressing ganglion cells. Thereby, the amount of light that reaches the retina is even more reduced (Thyagarajan et al., 2010).

Another important step towards the clinical application of ChR2 for vision restoration therapies is a successful translation of ChR2 delivery, and functional as well as safety tests in nonhuman primates. To this end, ChR2 delivery to ganglion cells has already been established in marmosets, demonstrating the overall feasibility, although very few animals were tested (Ivanova et al., 2010). High ChR2 expression from the CAG and CMV promoter was detectable at peripheral regions, whereas the foveal regions were harder to target. ChR2-mediated light responses were assessed by electrophysiology, but sophisticated tests for immunogenicity have not yet been developed.

In order to deal with immunogenicity of microbial opsins in human patients, intrinsic opsins would be a way to circumvent potential immune responses. Richard Masland’s group has successfully tested melanopsin, the photopigment that is normally used by retinal ganglion cells for non-image-forming vision (Lin et al., 2008). When overexpressing it ectopically in ganglion cells, which do not normally express melanopsin, these cells became light sensitive and former blind rd1 mice responded to light stimuli in behavioral tests. However, two important drawbacks, which might get solved by melanopsin protein engineering, are the low temporal resolution (high latency) and kinetics (prolonged activity in the range of seconds). Melanopsin belongs to the group of GPCRs and therefore, the signals are amplified within the signaling cascade, suggesting that less light is needed for activation. Consequently, the functional activation of melanopsin in non-intrinsically photosensitive retinal ganglion cells implies the recruitment of intrinsic G-protein cascades downstream of the receptor protein: it is unknown which alterations to the default signal cascade occur. Despite the fact that it functionally works, more mechanistic insights are needed to rule out unwanted side effects in regular signal pathways. Also, an engineered DNA-encoded light-gated ionotropic glutamate receptor (LiGluR) has been developed and further improved for resensitizing blind retinas of mice and dogs (Caporale et al., 2011; Gaub et al., 2014). However, LiGluR functionally depends on a chemically engineered photoswitch that needs to be co-injected. Despite its advantages, this dual components approach would be more difficult to translate than standalone optogenetic proteins that use intrinsic retinal as a chromophore.

When targeting retinal ganglion cells, optogene expression is not limited to the eyeball; axons and their terminals are also decorated with light-sensitive molecules. Hence, even a mild immune response would not be constrained to the eyeball, which would be removable by surgery; it would also be present in higher brain areas. Therefore, a restricted expression by localizing sequences to the soma and dendrites of ganglion cells would be desirable. In addition, by localizing a hyperpolarizing tool to the dendritic tips, and a depolarizing optogene to the soma, it has been shown that antagonistic center-surround receptive field interactions could be mimicked (Greenberg et al., 2011; Wu et al., 2013). In this light, targeting ChR2 to ganglion cell axonal initial segments where action potentials originate can improve light sensitivity and alter firing patterns (Grubb and Burrone, 2010; Zhang et al., 2015). Hence, the addition of localization sequences to optogenetic tools further refines and improves their use for turning retinal ganglion cells into artificial photo-sensing cells in blind retinas.

## Targeting Inner Retinal Cells

The rationale for targeting inner retinal neurons, such as amacrine and bipolar cells is to preserve certain retinal processing features. In addition, optogene expression might be contained within the retina for safety reasons. We have learned that the injection of AAV particles into the vitreous to target ganglion cells also results in ChR2 expression in some amacrine cells, whereas gene delivery to bipolar cells was extremely inefficient. Nevertheless, the use of ubiquitous promoter elements in combination with *in vitreal* injection of AAV particles that by default efficiently transfect ganglion cells is suboptimal for two main reasons: first, the optogene responses in ganglion cells would mask or overwrite light responses from inner retinal neurons; second, the GCLs would act like a sponge and take away substantial amounts of viral particles before they reach inner retinal cells by diffusion. Applying cell-type-specific promoter elements and engineering viral capsids that don’t get caught by ganglion cell surface receptors would be a solution.

Indeed, Botond Roska’s group has shown that a small sequence from the mGLUR6 promoter region (Kim et al., 2008) was specific enough to restrict ChR2 expression in ON bipolar cells of normally blind rd1 mice (Lagali et al., 2008, Figure 7). This specific reactivation of the ON pathway in the absence of intrinsic photoreceptor input led to light responses in ON ganglion cells that were transmitted to the visual cortex, as measured by VEPs. These formerly blind mice were also able to perform significantly better in visually guided behavioral tests compared to blind littermates. It is of importance that some retinal processing features, such as feed forward and lateral inhibition, were restored: this demonstrates the overall feasibility of restoring retinal processing by optogenetic intervention further upstream in the retinal pathways. Nevertheless, this first cell-type-specific approach was restricted to *in vivo* electroporation for gene delivery (Matsuda and Cepko, 2007), a method not suited for optical gene transfer in patients. At that time, the default AAV capsids failed to get efficiently incorporated into bipolar cells independent of the injection site. Since then, extensive screens with mutated, and thereby modified, viral capsids have been performed for optimized transfection of mouse ON bipolar cells (Dalkara et al., 2013). The efficient ChR2 expression upon *in vitreal* administration of AAV particles has been achieved, and has even been further improved by using several repeats of the mGLUR6 promoter element (Doroudchi et al., 2011; Cronin et al., 2014; Macé et al., 2015). Also, improved ChR2 variants (Kleinlogel et al., 2011a) have been applied to ON bipolar cells via AAV gene transfer (Cronin et al., 2014).

**Figure 7:**
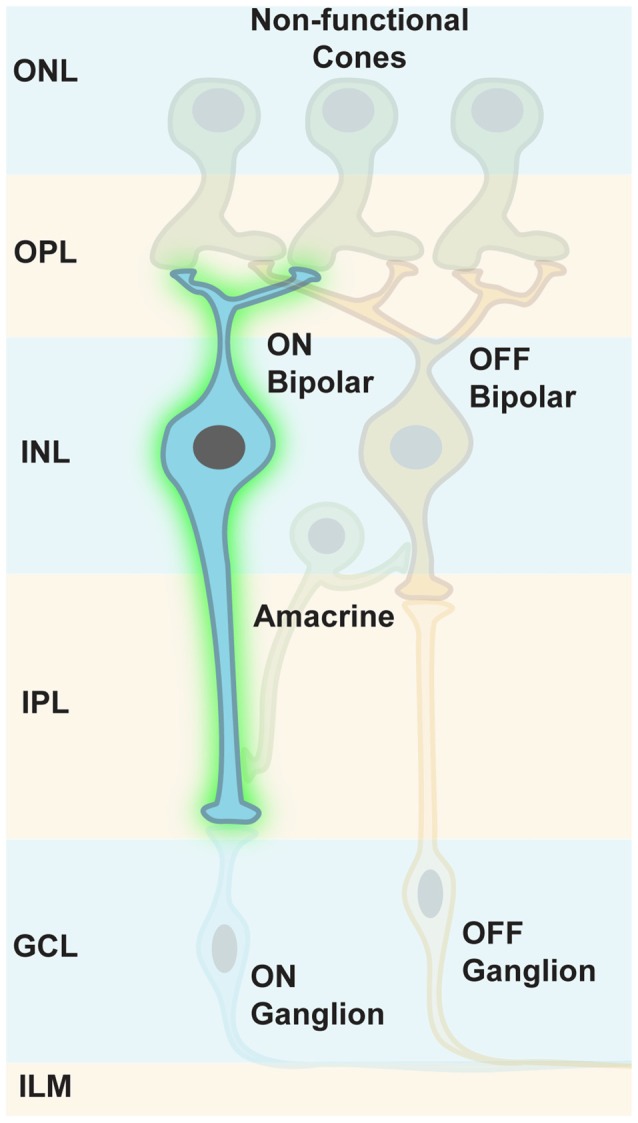
**Targeting ON bipolar cells.** Some retinal processing features, such as feed forward and lateral inhibition, can be restored when ON bipolar cells are optogenetically modified to respond to light. Intrinsic photoreceptor function is absent but not required.

To improve light sensitivity and reduce the potential immunogenicity of microbial opsins, intrinsic and engineered opsins have been applied to ON bipolar cells, similar to the techniques for ganglion cells. For example, a fusion protein from the intracellular domain of mGluR6 and melanopsin, called Opto-mGluR6 was about 4 log units more sensitive than wildtype ChR2 (van Wyk et al., 2015), likely because of the incorporation of a pre-existing G-protein cascade for signal amplification. The kinetics of Opto-mGluR6-mediated light responses in ON bipolar cells was comparable to normal light responses, overcoming the normal slow melanopsin kinetics. The regained visual information also resulted in better performances in visually guided behavioral tests. As Opto-mGluR6 was tailored to piggyback on the mGluR6-driven G-protein cascade, there are two reports proving that ectopical expression of human rhodopsin, the photopigment of rod photoreceptors, in ON bipolar cells leads to a restoration of light responses in formerly blind rd1 retinas (Cehajic-Kapetanovic et al., 2015; Gaub et al., 2015). Mechanistically, the recruited G-protein cascade members are currently unknown. The treated formerly blind retinas regained light-sensitivity with 200-fold lower intensity than ChR2: whereas ChR2 is only active over a ~2 log unit range of intensity, rhodopsin responses span over a 5 log unit range of intensities. Rhodopsin-mediated VEPs in formerly blind mice were detectable, and these mice also succeeded in visually guided behavioral tests. The light intensity needed for rhodopsin activation was similar to synthetic Opto-mGluR6. The regained responses of ganglion cells covered the full wildtype spectrum from excitatory to inhibitory, either being sustained or transient (Cehajic-Kapetanovic et al., 2015).

Taken together, the optogenetic toolbox for resensitizing ON bipolar cells is well equipped. The mGluR6 promoter element and its tandem version mediate sufficient and cell-type-specific transgene expression. Substantial AAV capsid modifications, followed by *inner vitreal* injections, have overcome inefficient transfection of inner retinal cell types. By this approach, about half of the retinal pathway, namely the ON pathway, can be restored, which subsequently leads to restored retinal signal processing and thereby, more aspects of the visual scene are sent, encoded as trains of action potentials, to higher brain areas. It is only a matter of time before specific OFF bipolar cell promoter elements are discovered, which would allow a spectrally-shifted optogene to be targeted to OFF channels. Thereby, both major retinal pathways would be restored, and would likely improve the quality of optogenetic vision. Alternatively, and if specific promoter elements become available, specific amacrine cells, such as the AII type could be targeted. Due to its specific connections, this would be able to drive ON and OFF ganglion cells separately by one depolarizing optogene. Of note, but not included here, there are also alternative photochemical reactivation approaches in blind retinas (Polosukhina et al., 2012; Tochitsky et al., 2014). Whenever photoreceptors in retinal degeneration diseases are lost, targeting inner retinal neurons with DNA-encoded light sensors is an attractive strategy to restore retinal processing features and overall visual function in formerly blind patients.

## Targeting Retinal Photoreceptors

**Figure 8:**
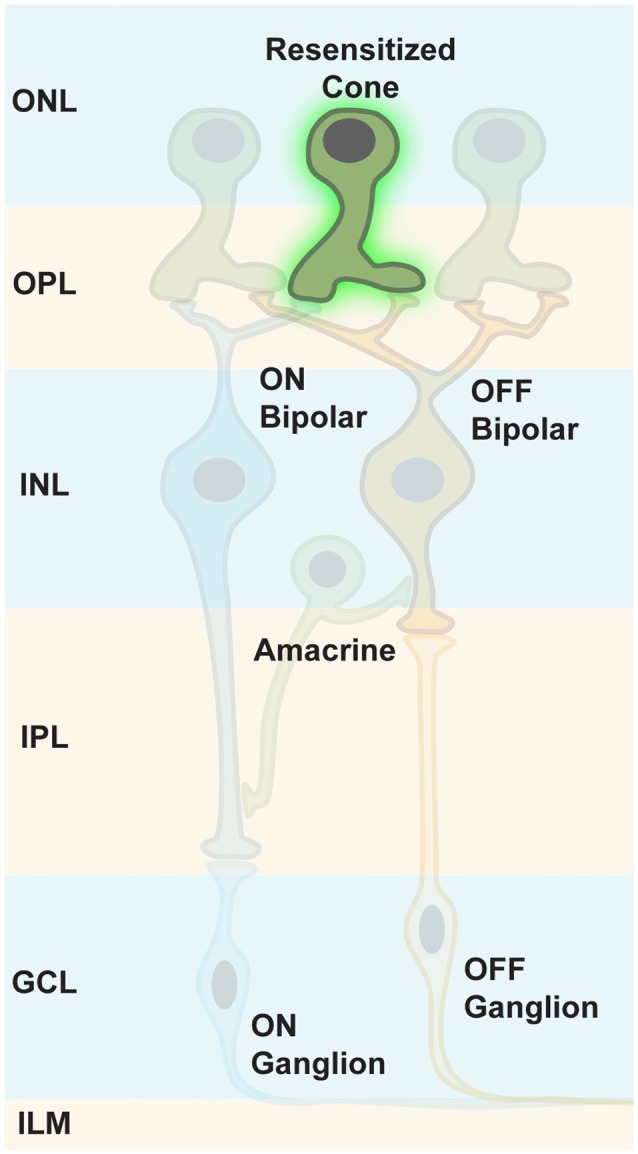
**Targeting persisting cone photoreceptors.** To maintain the maximum computational capacity of the retinal circuit, restoring photoreceptor function using optogenes is a valid option for RP patients whose cones are viable, but lack OS.

It has been thought that both photoreceptor types, rods and cones, die rather fast in retinal degeneration diseases, resulting in a retina completely depleted of photoreceptors. Therefore, the therapeutic window for targeting photoreceptors was thought to be very short. However, it has been shown that in RP, cones lose their light-sensitive OS and their cell bodies, then merge with the INL over time (Lin et al., 2009; Busskamp et al., 2010). Although transcripts of cone-specific markers were still expressed, the encoded proteins were not synthesized. Therefore, immunostainings for these markers did not stain, leading to the conclusion that cone photoreceptors had completely degenerated. In addition, some patients with late-stage RP had still areas with functional photoreceptors, or had cone photoreceptor cell bodies in the fovea (Lin et al., 2009; Jacobson et al., 2010). Therefore, the progression of complete photoreceptor loss appears to be more variable than previously believed, suggesting that there are patients with extended therapeutic windows for reactivating cone photoreceptors (Figure 8).

Photoreceptors can be easily targeted by AAV injections into the subretinal space, leading to a reversible detachment of the retina from the RPE (Bainbridge et al., 2008). Surrounding photoreceptors and RPE cells incorporate the viral particles. There are many photoreceptor-specific promoter elements for transgene expression restriction in rods and/or cones. Thereby, recombinant AAV genomes are not transcribed in RPE cells. The injection of liquids into the subretinal space is currently a standard procedure in laboratory settings for mice, rats, dogs, pigs and nonhuman primates (Li et al., 2008; Bruewer et al., 2013; Vandenberghe et al., 2013; Ye et al., 2016) as well as for clinical applications such as the RPE65 treatment in LCA (Bainbridge et al., 2008).

Resensitizing photoreceptor cells in diseases leading to blindness resemble a reactivation of retinal pathways leading to complete inner retinal networks, which can process the light information. Busskamp et al. (2010) of Botond Roska’s group applied an enhanced hyperpolarizing light-sensitive chloride pump, called eNpHR (Gradinaru et al., 2008), specifically to remaining cone photoreceptors in two RP mouse models, thereby restoring the ON and OFF pathways as well as retinal processing features such as direction selectivity. The expression of eNpHR was restricted to photoreceptors by using specific promoter elements. This was important, since eNpHR expression in downstream neuronal cell types would lead to inhibition, removing these cells from the visual pathways. As recorded by VEPs, the light information was sent to cortical areas, also resulting in sophisticated visually guided behavioral changes. The eNpHR expression was well tolerated in wildtype control mice: along with gaining their dichromatic vision, these mice experienced a sensitivity shift towards higher wavelengths, highlighting that the intrinsic phototransduction cascade and the ectopic optogene were both at work. There were no signs of any toxic eNpHR effects detectable. Furthermore, eNpHR was shown to hyperpolarize human photoreceptors in *post-mortem* retinal explants. The peak of eNpHR activity is at 580 nm, and photons with this wavelength cause less photochemical damage. The amount of light for a saturated eNpHR response was safe, according to European guidelines. The range of eNpHR activity spanned 2.3 log units of light. Further sensitivity improvements were achieved by applying more sensitive hyperpolarizing optogenes (Chuong et al., 2014). Based on the recent rhodopsin expression in inner retinal neurons, rhodopsin might also work in remaining cone photoreceptor cell bodies and would result in a higher sensitivity.

It is thought that resensitizing photoreceptors would restore a relatively high resolution. Normally, cones form stacks of cell bodies in the fovea but each of their OS serves as a light-sensitive antenna (Provis et al., 2013). Therefore, the sizes of the OS define the highest visual resolution. Since the cone OS are lost in RP, and eNpHR is expressed in the membrane of the entire cell body, its size defines the restored visual resolution. In addition, cone cell bodies in the same vertical stack would respond to identical stimuli. However, these theoretical calculations might need to be corrected when the first patients are treated and tested, and they are able to report their experiences.

## Conclusion

Depending on the disease state and which cell types remain, there are multiple therapeutic interventions possible. From a biophysical perspective, optimizing light sensitivity of microbial opsins is at its limits. However, increasing expression levels and cellular targeting, as well as piggy-backing on the intrinsic G-protein cascade for signal amplification, will improve light sensitivity. It will also be interesting to see how hyperpolarizing anion channels perform in photoreceptors or OFF bipolar and ganglion cells. All microbial optogenes lack the signal amplification generated by the phototransduction cascade and therefore, their sensitivity will not reach the levels of intrinsic opsins. Even harnessing G-protein cascades will not tune light sensitivity towards natural conditions because the special light-catching compartments of photoreceptors, the OS, are still missing. There are currently some ongoing efforts to regenerate these cellular compartments in order to restore intrinsic cone vision (Busskamp et al., 2014). In addition, combinations of optogenetic interventions with neuroprotective approaches (Sahel and Roska, 2013) might be essential when, despite restored function the degeneration still progresses as reported in LCA canine models (Cideciyan et al., 2013).

Under the current settings, the next goal is to restore monochromatic vision using optogenes. Ultimately, restoring color vision by spectrally shifted optogenes is theoretically possible, but technically extremely challenging. Medical devices, amplifying goggles (Cepko, 2010), will soon be on the market and enable visual perception by microbial optogenes under normal light conditions. Starting optogenetic trials with less sensitive optogenes could also be a safety feature. If resensitizing retinal cells in blind patients causes side effects such as headaches upon optical stimulation, it would be possible to simply remove the goggles to stop optogenetic visual activity.

In summary, there has been promising progress in the vision restoration field. The translation of optogenetic approaches has been thoughtfully discussed by experts (Francis et al., 2013). Of note is that the first clinical trial using wild type ChR2 was started end of 2015 (NCT02556736). The application of optogenes is a promising and mutation-independent approach to restoring vision. The contributions of the entire field are invaluable and it is very exciting that these therapeutic interventions will be implemented very soon.

## Author Contributions

SDK, AS, EB and VB commented and wrote the review article. The figures were made by SDK, AS and EB.

## Funding

This work was kindly supported by the following funding programs: a CRTD seed grant (SDK), a Volkswagen Foundation Freigeist fellowship (A110720; VB), and an ERC starting grant (678071—ProNeurons; VB). The Deutsche Forschungsgemeinsschaft (SFB 807) and the Max-Planck Society support to EB.

## Conflict of Interest Statement

The authors declare following patents related to ChR2 (WO2003084994 A3 and WO2012032103 A1 to EB) and to vision restoration approaches (WO2009127705 A1, WO2011039161 A1 and WO2015044890 A3 to VB). EB is a member of “Gensight Biologics” (France) scientific advisory board.

## Footnotes

^1^https://sph.uth.edu/retnet/home.htm
